# Endotoxin tolerance and trained immunity: breaking down immunological memory barriers

**DOI:** 10.3389/fimmu.2024.1393283

**Published:** 2024-04-29

**Authors:** Eduardo López-Collazo, Carlos del Fresno

**Affiliations:** ^1^ The Innate Immune Response Group, Hospital la Paz Institute for Health Research (IdiPAZ), La Paz University Hospital, Madrid, Spain; ^2^ Tumour Immunology Laboratory, IdiPAZ, La Paz University Hospital, Madrid, Spain; ^3^ Centro de Investigación Biomédica en Red (CIBER), Respiratory Diseases (CIBRES), Madrid, Spain; ^4^ Immunomodulation Laboratory, IdiPAZ, La Paz University Hospital, Madrid, Spain

**Keywords:** endotoxin tolerance, trained immunity, innate immune memory, monocyte, macrophage

## Abstract

For decades, innate immune cells were considered unsophisticated first responders, lacking the adaptive memory of their T and B cell counterparts. However, mounting evidence demonstrates the surprising complexity of innate immunity. Beyond quickly deploying specialized cells and initiating inflammation, two fascinating phenomena – endotoxin tolerance (ET) and trained immunity (TI) – have emerged. ET, characterized by reduced inflammatory response upon repeated exposure, protects against excessive inflammation. Conversely, TI leads to an enhanced response after initial priming, allowing the innate system to mount stronger defences against subsequent challenges. Although seemingly distinct, these phenomena may share underlying mechanisms and functional implications, blurring the lines between them. This review will delve into ET and TI, dissecting their similarities, differences, and the remaining questions that warrant further investigation.

## 1 Concepts and history

Long considered rudimentary remnants of evolutionary history, innate immune cells were once thought incapable of memory and solely reliant on a swift, non-specific response to infection. However, the remarkable complexity of innate immunity is patent, beginning from its ability to rapidly dispatch specialized cells to the challenged site, to initiate and amplify the inflammatory response through the release of diverse molecules, both mediators and effectors. These responses were even considered “non-specific” or “unspecific”. However, they are triggered after sensing defined pathogen-associated molecular patterns (PAMPs) or danger-associated molecular patterns (DAMPs) by pattern recognition receptors (PRRs) expressed by innate immune cells, conferring specificity (1). That way, recognition of viral PAMPs by endosomal receptors initiates interferon-dependent responses, while the recognition of fungi by members of the c-type lectin receptors (CLRs) triggers the phagocytic machinery and the production of reactive oxygen species (ROS) (2). Therefore, specific innate immune responses are generated depending on the recognized insult.

Along these lines, while memory generation has traditionally been credited to the adaptive immune system, findings in plant biology have challenged this notion. Plants, lacking an adaptive immune system, exhibit remarkable resistance to reinfection, proving that memory can exist independently of adaptive immune mechanisms (3). Moving on the evolutionary scale to invertebrates devoid of adaptive immunity, macrophages from *Drosophila melanogaster* fruit fly protect the animal from lethal *Streptococcus pneumoniae* infection after an initial sublethal exposition to this bacterium (4). Similar long-lasting memory processes have been also described for other invertebrates such as beetles, showing even transgenerational inheritance (5, 6). Turning to mammals, a growing body of experimental and clinical evidence since the early 20^th^ century has pointed to the existence of a form of memory that engages innate immune cells. This memory manifests as both enhanced and suppressed immune responses elicited by prior exposure to diverse stimuli, including whole pathogens, tumour cells and molecules derived from both.

In this context, two distinct phenomena, endotoxin tolerance (ET) and trained immunity (TI), have been described. ET, mainly characterized by a reduced inflammatory response following repeated exposure to a stimulus, primarily serves as a protective mechanism against excessive inflammatory reactions (7). In contrast, TI is associated with an enhanced immune response following prior priming, playing a critical role in mounting a robust proinflammatory mechanisms against subsequent challenges of the innate immune system (8).

On the one hand, probably one of the first solid observations of ET was reported by Paul Benson in 1947 when he induced lipopolysaccharide (the Gram-negative endotoxin, LPS) tolerance in rabbits by repeatedly injecting them with this endotoxin (9). In his words: “*Animals which received daily injections of pyrogens [LPS] for periods of several weeks showed no sign of deterioration in general health. They tended to gain weight, their coats remained sleek, and there was no special tendency to develop intercurrent infections*”. The phenomenon was also observed in humans recovering from malaria, who exhibited dampened fever responses upon re-challenge with endotoxin (10). The concept of ET was quoted in 1965 when describing a cross-tolerance effect in volunteers inoculated with *Plasmodium cynomolgi* via mosquito bites (11). These findings highlighted the existence of cross-reactivity among various stimuli in ET. In this line, a ground-breaking study published in 1969 demonstrated that prior exposure to live *Salmonella typhosa* exhibited the remarkable ability to dampen the inflammatory response triggered by endotoxin or killed bacteria (12). However, prior to this study, some clinical examples of ET were documented in patients with pyelonephritis (13) and those recovering from typhoid or paratyphoid fever (14). These reports further emphasized the widespread presence of ET in human health. Subsequent studies in mice revealed that sublethal doses of LPS conferred protection against subsequent lethal LPS challenges (15). It is worth noting that a large number of studies indicate a critical role for the monocytes/macrophages (MΦs) in ET (7, 16–18). In fact, both murine and human MΦs exhibit reduced inflammatory responses upon endotoxin challenge if they have been previously exposed to endotoxin (19).

On the other hand, initial observations of TI effects emerged in 1934, spearheaded by the Swedish scientist Carl Näslund, when he realized that the protection achieved following vaccination of neonates against tuberculosis with bacillus Calmette-Guérin (BCG) was beyond the decrease of deaths due to *Mycobacterium tuberculosis* infection. At that time, Näslund talked about “nonspecific immunity” (20). Similar processes were later observed in 1978 when BCG was shown to confer protection against parasitic *Schistosoma mansoni* (21) and *Candida albicans* fungal infections in 1992 (22). Remarkably, this observation was done in athymic nude mice lacking adaptive immunity, pointing to innate effectors as lead mediators of the protection. A 1978 study demonstrated similar protection against *Staphylococcus aureus* using prophylactic administration of fungal β-glucan (23). Interestingly, a 1986 study observed protection against a virulent form of *Candida albicans* following infection with a non-pathogenic strain (PCA-2), suggesting cross-protection within fungal pathogens (24).

It was in 2011, almost a century after the Näslund’s seminal report, when Mihai Netea began to name this type of memory process as innate TI (25). Nowadays, we know that TI at least contribute –if not lead– heterologous protective effects observed after vaccination campaigns such as protection against malaria (26), unrelated respiratory tract infections and sepsis (27) following the administration of the BCG vaccine. Another example comes from children receiving the oral polio vaccine (OPV), who, as expected, get protection not only against poliomyelitis, but also against respiratory viral infections (28).

Based on these extensive experimental and epidemiological data, we propose a shift from the term “nonspecific immunity” to “heterologous immunity”. As we will discuss later, the term “heterologous immunity” better reflects the specific receptors, signalling pathways, and molecular mechanisms that are involved in the development of these memory responses.

## 2 Endotoxin tolerance: beyond inflammation’s realm, yet not an immune paralysis

Sepsis, in which endotoxin tolerance is patently observed, serves as a clinically relevant example of ET. This complex condition arises from an uncontrolled inflammatory response by the innate immune system following a systemic infection. The evolution of ET in sepsis involves two phases. Initially, patients experience an exaggerated inflammatory response that progresses to a modulated state (7, 29, 30). According to several authors, during the second stage of sepsis the innate immune cells of these patients exhibit a patent ET (7, 18). This condition is clinically associated with an increased risk of secondary infections and mortality (31).

While the main hallmark of ET is undoubtedly a reduced inflammatory response, this phenomenon is not merely a suppression of pro-inflammatory cytokines production. In fact, sepsis monocytes treated ex vivo with LPS exhibit a diminished expression of costimulatory molecules like CD80, CD40, and HLA-DR, compromising their antigen-presenting capabilities (32–34). This reduced antigen presentation is evident in mixed lymphocyte reactions, where LPS-treated sepsis monocytes induce significantly low T cell proliferation (32, 35, 36).

Furthermore, sepsis monocytes show enhanced phagocytic activity, demonstrating a heightened ability to engulf and eliminate pathogens. This enhanced anti-microbial activity is also reflected in the ability of supernatants from sepsis monocyte cultures to restrict bacterial growth (32). Consistent with these findings, LPS-treated sepsis monocytes express elevated levels of the antimicrobial gene HAMP (32). In addition to the phagocytosis activity, these cells promote tissue repair and remodelling (32). Together, all these characteristics define the complex interplay of immune modulation and pathogen clearance during an ET reported not only in the context of sepsis, but also in cystic fibrosis, stroke, cancer, heart infarction, and COVID-19 (37–42). The main reported inducers of endotoxin tolerance in these contexts, to our current knowledge, are LPS, mitochondrial DNA (mtDNA), Pellino-3, lipoteichoic acid, and erythropoietin (EPO) (9, 40, 43–45).

### 2.1 Molecular mechanisms implicated in endotoxin tolerance

Intriguing studies of ET development in gene-deficient mice have shed light on the participation of intracellular molecules, including SHIP-1, A20, SOCS-1, TREM-1 and IRAK-M (7, 46–48). Among all of them, the pseudo kinase IRAK-M –also known as IRAK-3 from the interleukin-1 receptor-associated kinase (IRAK) family–, stood out as a potential master regulator of ET (16, 42, 48–50). In fact, its expression is consistently induced during ET and has been linked to a range of human pathologies, including sepsis, cancer, acute coronary syndrome, and asthma (51). Additionally, published data indicate that IRAK-M is not expressed in myeloid cells under steady-state conditions but is rapidly induced by the initial LPS challenge (16). Structural analysis and indirect evidences suggest that IRAK-M regulates the LPS response by inhibiting the downstream signalling pathway of toll-like receptor 4 (TLR4) (52). However, because not all ET characteristics are controlled by IRAK-M expression, other master regulators should be considered.

Despite evidences from conditional genetically ablated mice demonstrating HIF1α role in promoting myeloid cell-mediated inflammation and pro-inflammatory gene expression (53, 54), a meta-analysis of sepsis leukocyte datasets uncovered a strong correlation between elevated HIF1α and IRAK-M levels in independent cohorts of sepsis patients exhibiting the hallmarks of ET (32). This correlation suggests a more nuanced role for HIF1α in ET, where it not only upregulates IRAK-M expression in monocytes but also concurrently downregulates pro-inflammatory cytokine production and monocyte reprogramming (44), leading to the immunosuppressive phenotype and enhanced protective functions characteristic of ET such as phagocytosis, anti-microbial activity, and tissue remodelling (32, 37). This seemingly paradoxical effect stems from the complex temporal dynamics of HIF1α activation in human monocytes. Initially, its activation triggers a pro-inflammatory program, orchestrating a potent immune response. However, chronic activation paradoxically flips the switch, inducing negative regulators like IRAK-M, which ultimately dampen inflammatory responses and lead to an immunosuppressive phenotype. This shift towards immune suppression phenotype is crucial for ET.

In addition, the expression of the programmed cell death-1 ligand (PD-L1) has been described during ET in sepsis (36, 55) and COVID-19 patients (37, 56). Of note, both PD-L1 blocking and knockdown assays on tolerant monocytes from both patients with sepsis and the *in vitro* model reverted the impaired adaptive response observed during ET. Mechanistically, HIF1α translocates into the nucleus and drives PD-L1 expression during ET in human monocytes (36, 37, 55–57).

Interestingly, repeated endotoxin exposure reprogrammed monocytes at the chromatin level, with 80% of accessible regions becoming more open (17). This resulted in altered gene expression, primarily towards genes involved in detoxification and responding to environmental stress-induced cell damage. Notably, increased expression of metallothionein genes, key players in both natural and xenobiotic heavy metal detoxification, emerged as a potential biomarker of an immunosuppressive state (17). Additionally, it has been reported that the NFkappaB/IkappaB pathway plays a role in regulating gene expression during endotoxin tolerance (58).

Finally, microRNAs, short non-coding RNA molecules (~22 nucleotides), have also emerged as critical regulators of inflammatory responses during ET. Numerous studies have implicated several microRNAs in ET development, including miR-98, miR-125b, miR-132, miR-146a, miR-155, miR-221, miR-222, miR-579, and the let-7 family (59–63). However, the potential impact of miRNA dysregulation on the development and progression of inflammatory diseases warrants further investigation.

### 2.2 Long-lasting effect of endotoxin tolerance

One of the most important issues in a phenomenon involving a memory is its duration. Although there are not a significant number of studies that address the issue, in the case of ET, *in vitro* experiments have indicated that the ET hallmarks are fully kept for up to 5 days (42). It is only after this period of time that human monocytes “forget” their previous encounter with the endotoxin, but considering their relatively short lifespan they cannot be functionally available for much longer.

Interestingly, even a short-term (around one hour) exposition to an endotoxin such as LPS is sufficient to induce some level of ET in human monocytes. The refractory state is more evident when the first endotoxin challenge lasts for 6 – 8 h (42). Importantly, it has been observed that the ET does not influence cell viability, suggesting that monocyte counts could be normal during an infection induced ET state, but a significant proportion of these cells would not be functional.

In line, an ET state in circulating monocytes isolated from septic patients correlates with longer stays in intensive care units, prolonged mechanical ventilation, and a higher incidence of re-infections (64). Moreover, monocytes isolated from patients who went on to survive their septic episode were found to regain endotoxin responsiveness, whereas normal reactivity was never restored in non-survivors (65). Thus, the phenomenon of ET is thought to play an important role in the susceptibility to reinfection in patients with severe sepsis.

Moving to an *in vivo* model, always in humans, the duration of ET differs from what has been reported *in vitro*. In 12 healthy male non-smoking volunteers who received LPS twice, the *in vivo* cytokine response following LPS administration is still impaired after two weeks (57).

These discrepancies in ET duration between *in vitro* and *in vivo* models may have their origin in the involvement of tissue-resident cells whose effect is lost in an *in vitro* approach. In any case, this is a question to which no robust answer has yet been given.

## 3 Trained immunity: priming to boost inflammation

TI involves the long-term reprogramming of innate immune cells, resulting in an altered pro-inflammatory response to subsequent challenges, whether related or unrelated to the initial trigger. This concept aligns with the phenomenon of ET, where a first encounter with specific signals primes the immune system for a tailored response in later stages (66). β-glucans from the cell walls of fungi such as *Saccharomyces cerevisiae* and *Candida albicans*, and also the tuberculosis vaccine BCG, are the best characterized agents inducing TI (67–70). The priming of peripheral blood mononuclear cells (PBMCs), purified monocytes, neutrophils, or natural killer cells (NKs) with any of these TI inducers leads to a boosted inflammatory response upon a secondary stimulation with either the same or heterologous agents. Examples of such heterologous stimuli, considering the fungal or mycobacterial origin of the TI inducers, are the TLR2 ligand Pam3Cys, the TLR4 ligand LPS and whole microorganisms such as Gram-negative bacteria *Escherichia coli* or *Bacteroides fragilis*, Gram-positive *Staphylococcus aureus* or alternative mycobacteria such as *Mycobacterium tuberculosis* (67–70).

Of note, the portfolio of compounds inducing TI has notoriously increased since its initial formal definition, extending to alternative TLR ligands including polyI:C (TLR3 ligand) (71) and flagellin (TLR5 ligand) (72), fungal chitin (73), to more complex agents such as heat-killed mycobacteria (74, 75) and western diet (76). The capacity of this last TI inducer seems to rely on endogenous oxidized low-density lipoprotein (oxLDL) (76, 77) generated because of the hypercaloric diet intake, leading to the notion that also “self” assets such as certain DAMPs can induce TI (78). This is the case for uric acid (79), vimentin (80) and even hyperglycaemia (81).

As indicated previously, some vaccines show heterologous protective effects beyond the specific pathogen against which they were developed. Due to that, these vaccines can also be considered TI inducers. BCG is the prototype under this statement, but induction of TI by alternative vaccinations using attenuated agents such as *Influenza* (82) or *Vaccinia* (83) has also been experimentally demonstrated. Interestingly, not only the active vaccine agent can induce TI, but also some adjuvants included in vaccine formulations can generate this boosted inflammatory response (84). For instance, the oil-in-water emulsion adjuvant AS03 included in GlaxoSmithKline’s H1/N1 and H5N1 *Influenza* vaccines (85), and the lipid nanoparticle carrier employed for mRNA vaccines against COVID-19 (86). Under the light of the dual impact of these formulations in both innate and adaptive immunity, the concept of TI-based vaccines (TI-bV) (87) is gaining attention as vaccines designed to cover a wider spectrum of pathogens beyond antigen-specificity. An example of TI-bV is the inactivated polybacterial mucosal vaccine MV130, a mixture of whole heat-inactivated bacteria that, by inducing TI, confers heterologous protection against fungal and viral infections (88, 89).

While ET dampens the immune response, TI presents a contrasting picture. Though marked by an initial surge in cytokine production, the hallmark of TI lies in a broader reprogramming of the pro-inflammatory response. This reprogramming yields diverse outcomes depending on the specific immune cell type under study, including elevated levels of activation markers such as CD11b in monocytes (90) and neutrophils (70), Iba-1 in microglia (71) or scavenger receptors (CD36, SR-A) in macrophages accompanied of overproduction of ROS (70), as well as increased uptake of particles and phagocytosis (77, 91). This last feature is intriguing because as indicated before, ET- monocytes show enhanced phagocytic capacity.

All these events at the cellular level are reflected experimentally in heterologous protection against a plethora of challenges, both infectious and tumour related. Thus, prophylactic TI induction by systemic administration of β-glucan or BCG confers protection against bacteria including *Staphylococcus aureus* (92), *Listeria monocytogenes* (93) or *Mycobacterium tuberculosis* (94). Heterologous protection is also induced against fungal *Candida albicans* (68, 69), parasites such as *Leishmania braziliensis* (95) and viral infections including SARS-CoV-2 (96). Of note, this heterologous protection is also noticed against cancer development, both in grafted primary tumours (97, 98), and spontaneous (99) tumour models. Importantly, some of these experiments have been performed in SCID, NSG and mice lacking adaptive immunity *Rag1*-deficient (68, 69, 98), indicating that the observed heterologous memory process relies exclusively on innate immune cells.

Despite the diversity of the TI-related processes described above, there are some shared common mechanisms leading to the hallmark overproduction of cytokines characteristic of TI: the epigenetic and metabolic rewiring.

### 3.1 Epigenetics and metabolism take the lead of trained immunity

Priming stimuli leading to TI generate changes in the targeted cells allowing to perform a boosted inflammatory response upon secondary challenges. A good corpse of experimental evidence indicates that a deep epigenetic and metabolic reprogramming takes place in these settings.

Early mechanistic observations showed that β-glucan altered substantially the epigenetic landscape of trained monocytes, increasing notoriously the trimethylation of lysine 4 (H3K4me3) and acetylation of lysine 27 at histone 3 (H3K27Ac) (92). Importantly, these changes were found six days after β-glucan stimulation and prior to any secondary stimulation. The functional impact of these changes was revealed by killing the trained response using chemical inhibitors of enzymes generating those epigenetic changes, such as histone methyltransferases, while inhibition of histone demethylases showed no effect (69). These modifications poise specific promoters and enhancers in a more accessible conformation for transcription factors activated upon secondary stimulation.

Cellular metabolism is also profoundly affected upon TI induction. Glycolysis and the Krebs cycle are activated following both β-glucan and BCG stimulation, generating among others, lactate, acetyl-CoA and fumarate as key factors for the trained process (100, 101). Lactate acidificates culture media and can be quantified as a readout of TI, and the Extracellular Acidification Rate (ECAR) as a subrogate of the glycolytic metabolism (92, 100). Acetyl-CoA serves as a cofactor for chromatin-modifying enzymes involved in the above-mentioned epigenetic rewiring (102), as well as initiating platform for the cholesterol synthesis, also required for TI induction (101). Finally, fumarate induces TI by itself, mostly contributing to the enrichment of H3K27Ac residues at the epigenetic level (101). Nonetheless, mitochondrial oxidative phosphorylation is also activated following β-glucan-induced training, with implication in epigenetic remodelling through specific methyltransferases (103). Altogether, the interaction between epigenetics and metabolism establishes a crosstalk between each process, as genes encoding enzymes involved in the glycolytic pathway are among the transcriptionally open clusters primed upon training, and reinforcing the feed-back loop, metabolites generated during this metabolic rewiring modulates chromatin modifications (92, 102). Of note, these metabolic mechanisms have certain specificities that should be considered depending on the TI inducer (*e.g*., β-glucan or BCG) (100, 101).

The expression of specific long-noncoding RNAs (lncRNAs) adds another layer of complexity in this intersection as they facilitate epigenetic modifications by providing scaffolding for methyltransferases (104, 105), while the microARN mirR-9-5p is also implicated in the regulation of the immunometabolism and epigenetic rewiring triggered upon TI (106).

### 3.2 Molecular mechanisms implicated in trained immunity

Specific signalling pathways are triggered following the recognition of the different agents inducing TI. To date, the C-type lectin receptor Dectin-1 (69) and the intracellular NOD-2 receptor (90) are the best-described sensors triggering trained responses by most of the indicated inducers. Among the different signalling pathways triggered downstream Dectin-1 (107), the induction of TI relied on the Raf-1 kinase (67), while NOD-2 was coupled to Rip2 kinase (90). Of note, HIF1α following PI3K activation performs as a master regulator of trained processes triggered by different inducers (92, 101). Nonetheless, the implication of alternative signalling modules have been described for certain TI inducers such as polybacterial immunotherapies requiring Syk and MyD88 (108) or oxLDL (77) through liver X receptor (LXR), as well as specific LXR agonists (109, 110). Therefore, comprehensive mechanistic studies are required to identify core mediators of TI applying to the widest panel of inducers. At the same time, the characterization of stimuli-specific pathways leading to trained responses would allow their handling under clinicopathological situations of interest.

Of note, from a biological and structural point of view, it is hard to find common features between the already indicated TI inducers. Nevertheless, looking for common denominators, shared signalling pathways initiated by the main receptors involved in the recognition of these inducers could shed some light on the driving forces leading to TI induction. In this sense, Dectin-1 and NOD are pleiotropic receptors with the capability of sensing a plethora of ligands (107, 111). Signalling pathways triggered downstream these receptors are known to converge on RIPK2 upon diverse conditions (112, 113), as well as following TLR activation (114). However, the specific role of RIPK2 on TI induction has not been addressed. Therefore, it deserves future studies considering the central role that this kinase is achieving as a target for the treatment of inflammatory diseases (115).

Following this line, negative regulators of TI have been also identified. Trained responses were dampened upon SHIP-1 specific depletion in myeloid cells or its chemical inhibition (116). The activity of the isocitrate dehydrogenase 3α (IDH3α) was shown to inhibit HIF1α, leading to reduced trained responses by impacting both the metabolic and epigenetic reprogramming of β-glucan-trained monocytes (106). Another example is the expression of the Immune-responsive gene 1 (IRG1) leading to the production of itaconate and a consequent limitation of TI (117). Notably, all these pathways could serve to regulate the balance of the TI - ET equilibrium.

Looking at TI inducers, most of them are particles. This raises the question of the extent to which phagocytosis is involved in the trained process, not only as a readout (91), but also as a part of the mechanism implicated in its triggering. Interestingly, corpse engulfment primes pro-inflammatory responses in macrophages from *Drosophila melanogaster* fruit-flies through specific signalling pathways (118). Not only phagocytosis, but also autophagy has been related to TI, although the actual role of this process is unclear. While inhibition of autophagy dampened BCG-induced TI (119), innate immune priming due to uric acid reduced the extent of autophagy in human monocytes (79). Therefore, the role of this phagocytic process in TI deserves further investigation.

### 3.3 Long-lasting effect of trained immunity beyond *in vitro*

Most of the studies addressing molecular mechanisms related to TI have been performed using *in vitro* cultured cells. Nonetheless, *in vivo*, trained responses are observed for months following the administration of the TI inducer (88, 120). Therefore, those mature already differentiated cells that face this priming are no longer alive upon secondary stimulation. For that reason, in addition to cell-autonomous mechanisms, it is known that the numbers of hematopoietic bone marrow-derived progenitors (BMPs) are increased after exposition to TI inducers. Furthermore, those progenitors experience transcriptomic and metabolic changes that contribute to maintain durable memory of newly generated innate immune cells upon a second stimulus (94, 121). This process of central memory imprinting is revealed when mice adoptively transferred with BMPs from trained animals are protected against heterologous infections (122). A step further related to the spread of TI is its transmission across generations, implicating that the progeny of trained mice shows traits of innate immune memory as well as heterologous protection against infections (123). How this memory is imprinted at the bone marrow niche is not fully understood.

One possibility is that the TI inducers reach the bone marrow, acting directly on the BMPs. This could be the case of BCG when administered systemically through the tail vein in mice (94). However, this is unlikely in the case of particle β-glucan due to its large size (124), or TI inducers administered through mucosae (74, 88). Nevertheless, experimental approaches to formally address this concern are insufficient. For instance, mesenchymal stem cells uptake the polybacterial MV130 following sublingual administration, modifying their inflammation pattern (125). However, whether this immunotherapy directly targets BMPs has not been addressed. Interestingly, direct stimulation of BMPs with Dectin-1 ligands and cultured in the presence of M-CSF leads to the generation of macrophages with a boosted proinflammatory profile (126), suggesting that the targeting of the hematopoietic cells imprints a trained phenotype. Indeed, Dectin-1 expression was required in adoptively transferred BMPs to generate β-glucan-trained macrophages in Dectin-1-deficient receptors (127), indicating a direct role for the β-glucan receptor in the training process. Still, the TI inducer has not been found in the bone marrow after its systemic administration.

Another option is the generation of molecular intermediates between the recognition of the TI inducer and the reprogramming of BMPs. IL-1β has been proposed as one of these intermediates, as IL-1Repector (IL-1R) knockout mice lost both the heterologous protection against *Mycobacterium tuberculosis* and the myelopoiesis triggered by the prophylactic administration of β-glucan (128). This was also the case when providing a soluble IL-1R antagonist (128). IL-1β has also been proposed as the intermediate mechanism responsible of bacteria-derived nanovesicles inducing TI (129). Nonetheless, alternative mediators such as GM-CSF and IL-3 have also been postulated as potential intermediates supporting a proinflammatory trained phenotype (130). However, an experimental limitation to obtain these conclusions is that most of the mediator-deficient models are already sensitive to the assayed secondary stimulations and infections, making difficult to draw clear cause-effect relationships. Therefore, whether IL-1β can be considered a master mediator of TI still needs a deeper understanding of the underlying mechanisms supporting different forms of inducing the trained phenotype.

## 4 Tolerance *versus* training, similarities, and differences

### 4.1 Molecular signalling pathways

Because both ET and TI share a large number of molecular pathways, and monocytes/macrophages are established as the crucial cells in both contexts, it is important to establish a close comparison between these phenomena, broken down by associated molecular pathways.

#### 4.1.1 HIF1α

Perhaps one of the most important unanswered questions is why, in both ET and TI, HIF1α activation is crucial. Apparently, ET is the opposite of TI, yet this transcription factor emerges as one of the master regulators for both (32, 92).

Disrupting this pathway, either directly, through its upstream mammalian target of rapamycin (mTOR) signalling, or by specifically depleting HIF1α in myeloid cells, significantly weakens the trained responses triggered by various TI inducers (74, 92, 131). In the case of ET, experiments blocking HIFα –either by siRNA or using inhibitors of its expression– have shown that key features of ET, including IRAK-M expression, increased phagocytosis and tissue remodelling in the context of sepsis (32, 36) and COVID-19 (37), are reduced or even disappear. In addition, EPO-induced ET was also mediated by HIF1α (45).

Therefore, despite its master role in both innate memory processes, there must be alternative pathways implicated, making HIF1α necessary but not sufficient, at least for TI, as the chemical activation of HIF1α leads to dampened cytokines production in response to LPS, resembling ET (32). Considering the metabolic, epigenetic, and signalling pathways triggered downstream of key receptors involved in TI induction such as Dectin-1, a plausible model emerges. This model suggests a “division of labour” where various processes are compartmentalized, followed by a positive feedback loop between them (105). This coordinated effort ultimately leads to a fully developed TI program. In this sense, the calcium-dependent pathway have been proposed to contribute to the trained process (104) through the induction of long non-coding RNAs required for the epigenetic modifications accounting for the long-lasting effect of TI (104). According to this working model, the calcium-dependent pathway would not participate in the metabolism-related modifications, supporting a “division of labour”. Nonetheless, a consequence of that epigenetic priming is the enhanced expression of enzymes implicated in the glycolysis (92), thus generating a positive feedback loop. Of note, this particular example needs further characterization as the trained process has been postulated to be Syk-independent downstream Dectin-1 (67, 69), while the calcium-dependent pathway relies on the Syk proximal kinase to get activated (107).

Still, another variable to consider analysing the role of HIF1α is the “steady-state” of the myeloid cell when facing the initial priming stimulus. This becomes flawless in the differential impact of garlic-derived extracts on the memory phenotype observed between monocytes from healthy volunteers and septic patients. This extract induced ET in healthy volunteers while boosted inflammatory responses were observed in septic patients, in a HIF1α-dependent manner (132).

#### 4.1.2 TLRs

Endotoxin-induced ET via TLR4 established the ground for innate immune memory. Nonetheless, alternative Toll-like receptors have been implicated in these processes. For instance, Poly I:C (TLR3 ligand) and CpG (TLR9 ligand) induce TI in neutropenic mice depleted of neutrophils based on an anti-Ly6G antibody (35, 71). Flagellin (TLR5 ligand) conferred heterologous protection against the Gram-positive bacteria *Streptococcus pneumoniae* when administered through the airway mucosae (72). In addition, the role of TLR4 in innate immune memory might be dual. Consecutive intraperitoneal injections of low-dose LPS for 4 days gave rise to systemic ET after the second LPS administration, while TI was induced at the same time point in the brain, indicating a tissue-specific pattern or dynamics (133). Therefore, different TLRs, despite sharing upstream signalling components such as MyD88 and TRIF (134–136), can generate either ET or TI, but the context must be considered to establish this balance as the generation of one or the other might depend on the cellular environment (lack of neutrophils), route of administration (mucosal) or tissue location (brain).

Focusing on TLR4 as master LPS receptor, the signalling pathways triggered following this recognition involve both MyD88 and TRIF, with the latter leading to type I interferon production (134). This rises to possibility that a differential or preferential activation of one or the other could conduct towards a trained or tolerant phenotype. Lack of TRIF or IFNβ blockade impairs ET triggered by lipid-A from *E. coli* (135). However, type I interferon signalling was required to induce TI in alveolar macrophages following intranasal exposure to LPS (137), and indeed, both IFNα and β prime proinflammatory responses to secondary heterologous challenges (138, 139), resembling TI. Therefore, conflicting data raise questions about the specific role of these pathways in triggering each program. Nevertheless, it’s likely there are additional regulatory mechanisms downstream of MyD88 that determines the shift towards ET or TI, such as the presence and activation of the pseudokinase IRAK-M, which might be essential for key features of ET to occur (32, 50). This could be the key to understand how a shared signaling pathway leads to such distinct immune responses.

Along these lines, the stimulation of mast cells with LPS leads to a reduced cytokines production upon secondary endotoxin challenge, reproducing ET. Nevertheless, when *C. albicans* was used as heterologous challenge, TNFα release was enhanced, reminiscent of TI (140). How this TLR4 stimulation gives rise to both training and tolerance depending on the second insult is not understood, but the ET phenotype was sensitive to histone deacetylases inhibition (140). These findings suggest a potential “double-edged sword” role for TLR4, with its effect varying depending on the secondary stimulation. While its influence might differ across cell types, this possibility adds another layer of complexity to understanding TLR4’s involvement in this context.

#### 4.1.3 PI3K, Akt and SHIP1

PI3K and inositol phospholipid species generated by this enzyme are critical mediators of intracellular signalling pathways, including those triggered by myeloid receptors (141). Thus, different stimuli inducing ET such as low dose of LPS and chemical activation of the key glycolytic enzyme Pyruvate kinase M2 (PKM2) are accompanied by a repression of the PI3K/Akt pathway (140, 142, 143). Additionally, the expression of IRAK-M, critical mediator of cytokine downregulation during ET, was boosted upon PI3K inhibition (16). On the other hand, PI3K is strongly activated following β-glucan stimulation through Dectin-1, and its chemical inhibition dampens TI (92). Therefore, it is tempting to venture that this pathway establishes a balance between ET and TI, favouring the training process. In this regard, we speculate that the activation or non-activation of IRAK-M controlled by both HIF1α and PI3K may be crucial for the establishment of ET or TI.

As a fundamental signalling for cell biology, PI3K activity is tightly regulated (141). In line with a dampened PI3K activity required for the generation of ET, the expression of SHIP-1, a PI3K antagonistic phosphatase, was induced in peritoneal and splenic macrophages upon ET (46), being critical for the development of the tolerant process (144). Consistently, SHIP-1 depletion in myeloid cells or its chemical inhibition boosted β-glucan-induced trained responses (116).

However, as in many other examples all along this review, there are exceptions to this apparent rule. EPO induces ET in macrophages through PI3K/Akt activation as well as IRAK-M induction. That way, EPO administration confers protection against *Escherichia coli*-induced endotoxin shock by dampening proinflammatory cytokine production, accompanied of a reduced bacterial burden (45). Interestingly, this work proposes an independent induction of the PI3K/IRAK-M and HIF1α axes that could explain the appearance of concurrent ET and TI features due to the uncoupling of these two key pathways. Importantly, secondary stimulation is required to reveal the tolerant and/or trained phenotype, showing a dominant outcome. This raises the concern that most of current studies, while focusing on specific secondary stimuli, might overlook crucial mechanistic clues underlying the interplay between ET and TI. Conducting parallel experiments in both ET and TI contexts, evaluating responses to common heterologous challenges, could illuminate the molecular-level interactions between both processes and provide a more comprehensive understanding.

### 4.2 Chromatin modification, gene and metabolic reprogramming

Considering the divergent phenotypes, one could conclude that ET and TI are essentially two alternative phenotypes of the same process, namely, an innate reprogramming characterized by either reduced or enhanced inflammatory responses, two sides of the same coin. If that’s true, the two processes should share common driving mechanisms. However, this is not always the case, raising the question of till which extent there are certain specificities.

For instance, an interesting study addressed the heterologous memory effects of two viruses used as vaccines (*Vaccinia*) or vaccine vector (modified *Vaccinia* Ankara, MVA). In human primary monocytes, the former induced TI while MVA generated ET, analysed in terms of TNFα and IL-6 production upon secondary heterologous stimulation with LPS or Pam3Cys. Interestingly, the tolerant state was abolished in the presence of the histone methyltransferase inhibitor MTA, despite MVA did not alter the enrichment of the H3K4me3 residue on the promoters of TNFα and IL-6 (83). Hence, these results suggest that although induction of ET in these settings relies on epigenetic reprogramming, different epigenetic modifications than the conventionally observed upon TI take place.

Therefore, wide-ranging comparative studies at the epigenetic level are required to identify an essential chromatin accessibility program considering different genetic locations (promoters, enhancers) (17) to determine the core transcriptional response activated by diverse innate immune stimuli triggering either ET or TI. In this line, the pioneer study performed by Ruslan Medzhitov and collaborators in 2007 identified “tolerizeable” versus “non-tolerizeable” genes following LPS-induced ET in bone marrow-derived macrophages (145). A comparable study was conducted by Mihai Netea and colleagues in 2012 using purified human monocytes instead (92), where “trainable” versus “non-trainable” genes could be identified, although these terms were not used in this work. Studies using the same cell type and diverse inducers of innate immune memory as well as secondary stimulations are required to clarify the “black or white” paradigm between ET and TI, experiments that should be accompany of metabolic and phenotypic analyses. Good examples of these kind of projects are the epigenetic studies performed by Hendrik Stunnenberg and colleagues where describing differential epigenetic traits imprinted in ET and TI monocytes (146), demonstrating that ET can be reverted by β-glucan, yet whether the process works in the opposite direction was not addressed (147).

As metabolic reprogramming is also critical for the induction of innate immune memory, these processes need to be tightly regulated between ET and TI, in coordination with gene expression and epigenetics. The relevance of metabolism in this balance was apparent in leukocytes rendered tolerant following LPS exposition or after isolation from patients with sepsis. These cells showed a generalized metabolic defect at the level of both glycolysis and oxidative metabolism, which was restored after recovery of the patients (148). Similar results were obtained in monocytes isolated from healthy individuals after receiving a controlled intravenous dose of LPS (149). On the other hand, the activation of glycolytic metabolism is essential for the induction of TI (100, 101). A potential master regulation of this metabolic balance is the itaconate pathway linked to the expression of immune-responsive gene 1 (IRG1) (117). Both factors are induced upon LPS-induced tolerance and repressed by β-glucan, illustrating the equilibrium between both memory processes. To fully understand this interconnection, the role of alternative common regulators, and the effect of the primary stimulus on the recovery of cellular metabolism deserves further investigation as key determinants for the response to secondary stimuli.

### 4.3 Role of IL10 *versus* IL-1β

Several studies have indicated a promising role of soluble mediators for the development of ET. Among them, the immunomodulatory cytokine interleukin-10 (IL-10) plays a paradigmatic role. Neutralizing IL-10 using blocking antibodies during the first LPS stimulation avoids the expected reduction of TNFα production in response to a subsequent endotoxin challenge (150). However, since last century we know that IL-10-deficient mice still develop ET (150).

In line with, and despite of its profound anti-inflammatory role, IL-10 does not play a major role in regulating TI as its production is not modulated upon trained responses (67, 74). This could be relevant for the lack of deleterious inflammatory responses during TI or TI-bV (87).

Alternatively, IL-1β is profoundly modulated in both trained and tolerant processes. Of note, IL-1β is considered a key systemic mediator for TI induction following training with different inducers (128, 129). As IL-1β is also produced in response to LPS, this raises the question about the specific role of this cytokine for both ET and TI. Also and as described above for HIF1α, till which extent IL-1β is necessary but not sufficient for TI induction, as the mechanistic studies published in this sense use IL-1R-deficient mice or IL-1R antagonists (128, 129), instead of IL-1β supplementation.

Among the members of the IL-1 family, IL-37 deserves attention when talking about ET and TI. This cytokine has been described as an inhibitor of TI, consistent with its anti-inflammatory role (151). Interestingly, IL-37 is expressed at high levels in septic patients, acting as a mortality risk biomarker (152). Therefore, this cytokine appears to play dual roles during innate training and tolerant processes, regulating the expression of IL-1β (153). Furthermore, IL-37 controls cytokine production by mechanisms beyond its recognition by the canonical receptor IL-1R8, working at epigenetic levels (154). As epigenetics is essential for innate immune memory, it would be interesting to address the differential role of this cytokine upon well-defined ET conditions.

### 4.4 Potential role of immune checkpoints

The discovery and characterization of immune checkpoints (ICs) has offered a new avenue for studying the potential role of cell-to-cell communication in a number of pathologies beyond cancer. These surface molecules might function as ligands for lymphocyte receptors, fine-tuning the duration and intensity of the adaptive immune response. Notably, ICs can act as both stimulators and inhibitors of immune responses. Among the inhibitory ICs, the B7 superfamily and programmed cell death–ligand 1 (PD-L1) proteins hold promise for therapeutic interventions in various clinical settings. Interestingly, a report has indicated that human blood sepsis monocytes under ET expressed high levels of PD-L1 on their cell surface via the induction of HIF1α, and governed the impaired induction of T-cell proliferation during ET (36). Additionally, another study identified ICs and Ig-like V-type receptors in several cell populations in peripheral blood from another ET-related disease, COVID-19 (37, 56). In fact, levels of ICs were associated with the risk of secondary infections, one of the known consequences of ET. In contrast to what has been described in ET and to our knowledge, a possible role of ICs during TI has not been reported.

### 4.5 Innate immune memory duration

When talking about memory processes, time matters. How long these phenomena stand for is relevant for reaching clinical applications. *In vitro*, ET lasted for 5 days following the first LPS encounter (42), and classical TI models are performed up to 7 days after priming (67, 116). *In vivo*, while ET lasts for two weeks (57), trained responses are observed months after the administration of the TI inducer (88, 94, 155), therefore, this long-lasting memory cannot be explained by molecular reprogramming of mature cells, in particular, innate immune cells, whose lifespan is limited following their activation.

Of note, metabolic and epigenetic imprinting of hematopoietic progenitors at the bone marrow, giving rise to trained cells upon secondary stimulation, sustain these durable memory phenomena (94, 121). Following experimental training *in vivo*, ex vivo differentiation of these progenitors into trained mature macrophages, or adoptive transfer experiments transmitting TI capabilities to recipient mice (88, 122), unequivocally demonstrate the functional reprogramming of these multipotent ancestors. In humans, both hematopoietic stem and progenitor cells and monocytes showed boosted inflammatory responses associated to TI-related epigenetic traits three months after BCG vaccination (155).

As early as 1985, cytologic alterations in the bone marrow of LPS tolerant mice were observed (156), suggesting that the hematopoietic niche could play a role. Induction of ET in rat bone marrow cells following TNFα infusion of the animals demonstrated the functional relevance of hematopoietic stem cells in the tolerant process (157), something further confirmed in mice after LPS administration (158). Along these lines, stimulation of hematopoietic and progenitor cells (HPSCs) purified from the bone marrow with TLR2 and TLR4 ligands generate macrophages with a dampened capacity to produce inflammatory cytokines. In contrast, HSPCs activation in response to Dectin-1 ligands such as depleted zymosan or *C. albicans* leads to the generation of macrophages that produce higher amounts of inflammatory cytokines following a secondary stimulation (126). Therefore, the training of both the tissue-resident myeloid compartment and the hematopoietic niche-located cells allows to differentiate central innate memory, as the one imprinted at the level of bone marrow, from peripheral innate memory, ascribed to already differentiated myeloid cells (8). An intriguing observation from *in vivo* experiments performed in mice is that normal responsiveness returns back only few days after the initial exposure to LPS (156, 158), while enhanced responses following exposure to TI inducers last for months (88, 94, 155). This opens the question whether the imprinting at the bone marrow progenitors’ level is comparable between ET and TI.

## 5 Clinical implications

The clinical implications of ET are highly dependent on the specific disease context and its intricate immune dynamics. It can be a double-edged sword, offering protection against excessive inflammation, but also potentially leading to immunosuppression and increased vulnerability to infections.

As we referenced before, there are several clinical contexts where ET have been reported. In the case of sepsis, the ET can lead to a weakened immune response, increasing susceptibility to secondary infections and potentially increasing mortality. In contrast, it can prevent excessive inflammation and tissue damage, which can also contribute to death in sepsis. The key lies in achieving a balanced immune response. Another example of ET-related disease is the stroke (38), where ET might also lead to an inadequate immune response against post-stroke infections, increasing the risk of complications. On the other hand, this status could protect patients against excessive neuroinflammation, a major contributor to stroke damage. In COVID-19, its complex immunopathology involves both hyperinflammation and immunosuppression (159). Endotoxin tolerance might contribute to the latter in some cases, leading to higher viral loads and poorer outcomes. Finally, in the ET-related disease cystic fibrosis, chronic lung infections in patients involve complex immune responses, with potential implications for endotoxin tolerance (42). In this sense, more research is needed to understand how endotoxin tolerance affects the course of cystic fibrosis and whether modulating it could be beneficial.

On the other side, under any pathophysiological condition where pro-inflammatory responses can be considered as protective, the induction of trained immunity could be desirable. This mostly applies to infectious and tumour processes. However, despite a type-1 inflammatory reaction seems mostly convenient against infections, for instance, helminths are better fighted mounting Th2 responses (160). Whether trained processes boost this kind of reactions is not fully understood in the state-of-the art. Nevertheless, the great advantage of interventions based on TI is that by generating wide pro-inflammatory responses, protection against a broad spectrum of foreign pathogens is expected. This is actually the notion supporting the concept of TI-bV (87). However, as indicated before, boosted inflammation is not always desired. Autoimmune conditions, atherosclerosis or organ transplantation (161, 162) demand the dampening of such pro-inflammatory environments to minimize tissue damage.

In this sense, it is important to highlight the prophylactic concept of TI-based treatments that grounds its potential application for new designs in vaccinology (66). In order to achieve an advantage over an infection, TI has to be already generated, meaning that the priming has to take place before the secondary challenge (163). The need for a first priming to generate the pro-inflammatory condition also represents a window of opportunity to brake undesired trained responses by targeting TI-related mechanisms in advance. This strategy has been proved successful in the context of organ transplantations (80).

Therefore, both ET and TI have a long track ahead in the clinical harnessing of inflammatory conditions. Understanding molecular and cellular mechanisms, both unique and shared between both processes is key for reaching medical applications.

## 6 Outstanding questions

Despite all the research summarised here on ET and TI (see **Table 1**), several questions remain open.

1. Perhaps the most interesting will be to reveal the mechanisms that favour one sort of innate response over another when they use molecular pathways that are, if not identical, then very similar. In particular, bifurcation from HIF1α activation should be studied in depth using parallel models of ET and TI.
2. Another question to be answered would be the possibility of reversal from one state to the other with the use of cross-inductors of ET and TI. This would be of enormous interest in the clinic to reverse/induce an ET or an TI depending on the context.
3. Following on from the mechanisms underlying both phenomena, it would be interesting to explore polarization at the level of the bone marrow. Memory has to be durable in time, nonetheless, till which extent mechanisms implicated in ET and TI are shared at the level of myeloid progenitors is ill-defined.
4. Uncovering the molecular pathways that govern both innate immunity memory processes will allow to understand how exclusive they are, and till which extent, ET and TI can be, at least partially, triggered simultaneously.
5. Finally, there is a great need to establish unequivocally with *in vitro*, *in vivo* models, and using patient samples, a robust comparison of the functional hallmarks of both types of innate response memory. Progress has been made in the case of ET but less so in the context of TI.

**Table 1:** Summary of key features of endotoxin tolerance and trained immunity.

	Endotoxin Tolerance	Trained Immunity
**Inducers**	Lipopolysaccharide (LPS) (9, 15) Mitocondrial DNA (mtDNA) (40) Pellino-3 (43) Lipoteichoic acid (44) Erythropoietin (EPO) (45)	β-glucan (69) BCG vaccine (70) oxLDL (77) LPS (133) polyI:C (71) Flagellin (72) Fungal chitin (73) DAMPs: Uric acid (79), Vimentin (80) Heat-killed mycobacteria (74, 75) *Candida albicans* (67) Polybacterial MV130 (88)
**Mechanisms Implicated**	HIF1α (32, 37) IRAK-M (7, 46) PD-L1 (36, 55) A20 (46) SOCS-1 (47) TREM-1 (48) TLR4/MyD88/TRIF (135, 136) PI3K/Akt (16, 45, 142, 143) PI3K/SHIP-1 (46, 144) miRNAs: miR-98, miR-125b, miR-132, miR-146a, miR-155, miR-221, miR-222, miR-579, and the let-7 family (59–63) Epigenetic control (17) NFkappaB/IkappaB (58)	Epigenetic control (69, 101, 103) mTOR (92) PI3K/HIF1α (92, 101) Dectin-1/Raf-1 (67, 69, 107) NOD-2/Rip2 (90) Glycolysis (100) mirR-9-5p (106) LXR (109, 110)
**Phenotype**	o Low pro-inflammatory response (7) o Increased cellular cleanup (32, 42) o Tissue repair focus (32, 42) o Ineffective antigen presentation (36, 42) o Impaired adaptive immunity (36, 42)	o High pro-inflammatory response (67–70) o Increased activation markers (71, 90) o Boosted ROS production (70) o Increased particle uptake (77, 91)
**Clinical Impact**	Sepsis (7, 16, 32, 64) Cystic Fibrosis (41, 42) Myocardial Infarction (40) Stroke (38)	Vaccination (87) Atherosclerosis (162) Organ transplantation (161)
**Long-lasting effect**	*In vitro* (human monocytes): 5 days (42) *In vivo* (humans): up to 14 days (57)	*In vitro* (human monocytes): 7 days (67) *In vivo* (humans): 3 months (155) *In vivo* (mouse): 6 - 7 months (94)

## Author contributions

EL-C: Conceptualization, Funding acquisition, Writing – original draft, Writing – review & editing. CdF: Conceptualization, Funding acquisition, Writing – original draft, Writing – review & editing.
